# Statistical modeling of health space based on metabolic stress and oxidative stress scores

**DOI:** 10.1186/s12889-022-14081-0

**Published:** 2022-09-08

**Authors:** Cheolgyun Park, Youjin Kim, Chanhee Lee, Ji Yeon Kim, Oran Kwon, Taesung Park

**Affiliations:** 1 Department of Statistics, Seoul National University, Seoul, Republic of Korea; 2 Department of Nutritional Science and Food Management, Ewha Womans University, Seoul, Republic of Korea; 3 Interdisciplinary Program in Bioinformatics, Seoul National University, Seoul, Republic of Korea; 4 Department of Food Science and Technology, Seoul National University of Science and Technology, Seoul, Republic of Korea

**Keywords:** Metabolic stress, Oxidative stress, Health space

## Abstract

**Background:**

Health space (HS) is a statistical way of visualizing individual’s health status in multi-dimensional space. In this study, we propose a novel HS in two-dimensional space based on scores of metabolic stress and of oxidative stress.

**Methods:**

These scores were derived from three statistical models: logistic regression model, logistic mixed effect model, and proportional odds model. HSs were developed using Korea National Health And Nutrition Examination Survey data with 32,140 samples. To evaluate and compare the performance of the HSs, we also developed the Health Space Index (HSI) which is a quantitative performance measure based on the approximate 95% confidence ellipses of HS.

**Results:**

Through simulation studies, we confirmed that HS from the proportional odds model showed highest power in discriminating health status of individual (subject). Further validation studies were conducted using two independent cohort datasets: a health examination dataset from Ewha-Boramae cohort with 862 samples and a population-based cohort from the Korea association resource project with 3,199 samples.

**Conclusions:**

These validation studies using two independent datasets successfully demonstrated the usefulness of the proposed HS.

**Supplementary Information:**

The online version contains supplementary material available at 10.1186/s12889-022-14081-0.

## Background

Lifestyle-related chronic diseases such as cardiovascular diseases (CVD), diabetes, hypertension, dyslipidemia, and obesity are heterogeneous and multifactorial [1]. These diseases resulted from sustained interactions between biological processes including antioxidant defense mechanisms and metabolic adaptation [2–5]. A comprehensive understanding of complex biological processes requires concurrent quantitative analysis of many individual components when defining an individual’s health and susceptibility to disease [1]. An accurate estimation of the current state and long-term prediction at an earlier life stage is essential to optimize health and alleviate the increasing burden on lifestyle-related chronic diseases [6].

A simple and effective visualization methodology may help to easily recognize current and future health status of individuals so that health behavior change can be made. The health space (HS) was conceptualized to statistically quantify individuals’ health status for assessing their responses in biological processes relevant to long-term health and disease outcomes by summing up the accumulated value of multiple biomarkers [7]. This HS can present a complex, multi-factorial health condition in a multi-dimensional space and visualize different groups of healthy and unhealthy individuals easily [8, 9]. Nevertheless, while this conceptual multivariate model was built in a few human intervention studies [9, 10], the methodology needs to be optimized and further validated in the general population with a large number of individuals.

The previous HSs simply included axes and points, and were only referring to approximate differences between groups, such as placebo and treatment groups. Although the points of different groups on the HS may seem to be distinct from each other, the groups may be in fact often overlapped excessively. As a result, they could not clearly distinguish the groups with different health status. Aiming to overcome these limitations, we propose a novel HS in two-dimensional space where the two axes represent oxidation and metabolism stress scores. We choose oxidative and metabolic stress because they are the main processes in which the imbalance can lead to various lifestyle-related chronic diseases [1].

In order to derive oxidation and metabolism stress scores and build HS, we first fitted three statistical models: logistic regression model, logistic mixed effect model, and proportional odds model. Second, we visualized an approximate 95% confidence ellipses of two scores in the HS representing the four distinct health groups. Third, we developed a novel index called the Health Space Index (HSI) which allows us to evaluate and compare the performance of the HS. HSI is a quantified measure representing how much the approximate confidence ellipse of each health status group are overlapped and provides information about the distinctness between groups on the HS. Additionally, to demonstrate the usefulness of the proposed HS, we performed simulation studies and validation studies on two independent cohort datasets. The proportional odds model showed the best power discriminating four health status groups.

## Methods

### Korea National Health And Nutrition Examination Survey data

We built the HS models using the Korea National Health And Nutrition Examination Survey 2007 − 2016 (KNHANES) data (32,140 samples) [11]. The surveys have been conducted by the Korea Disease Control and Prevention Agency (KDCA) for assessing the health and nutritional status of Korea since 1998. The survey collected approximately 10,000 individuals each year with information on socioeconomic status, health-related behaviors, biochemical and clinical profiles for non-communicable diseases [12]. From the data of individuals aged over 19 years old from KNHANES (*n* = 81,503), 49,363 samples were excluded for the following reasons: Aged less than 20-year-old (*n* = 26,768), missing information (*n* = 22,595) on anthropometric and biochemical measurements, disease, and smoking status. We then validated the HS models using two independent datasets. First, health examination dataset from Ewha-Boramae cohort with 862 samples were used as validation data. This data is from prospective cohort study of Korean male and female aged 19 year or above that underwent comprehensive annual or biannual health examination in Seoul National University Boramae Hospital (Seoul, South Korea) and analysis of biological samples was conducted at Ewha Womans University [13]. Out of a total of 1,464 participants, 602 samples were excluded due to missing information on history of disease, medication, and recommended food score (RFS). Second, population-based cohort from the Korea association resource project (KARE) with 3,199 samples were used. The cohort of KARE was established as part of the Korean genome and epidemiology study (KoGES) Ansan and Ansung study in which biannual repeated surveys were conducted in two provinces of South Korea. Physical examinations and clinical investigations were performed and measured, and anthropometric and clinical measurements were also obtained. [14]. Among 9,334 participants from 2001 to 2003, 6,135 samples having missing data on anthropometric and biochemical profiles, smoking, disease, and medication were excluded, leaving a sample of 3,199 participants.

For each dataset, we split the individuals into four health status groups: healthy group, a group with one metabolic risk factor, a group with two metabolic risk factors, a group with metabolic syndrome or oxidative stress-related disease group. Subjects diagnosed with any of the following diseases were categorized into the lifestyle-related chronic disease group related to oxidative and metabolic stress [2–5, 15, 16]: metabolic syndrome, diabetes mellitus, dyslipidemia, severe obesity, intermediate coronary syndrome, stroke, hypertension, and diet-related cancers (liver, colon, stomach, breast, prostate, and lung). In those datasets, age, sex (0 = male, 1 = female), WBC (× 10^3^ μL), GPT (μkat/L), smoking status (0 = never and past smoker, 1 = current smoker), BMI (kg/m^2^), Glucose (mmol/L), HDLC (mmol/L), and TG (mmol/L) were used. As the units of variables differed from one data to another, système international d’unités (SI) units [11] were adopted for modelling throughout the present work.

Our HS was constructed with two axes of oxidative and metabolic stress scores. Each score was derived from predictor variables with biological relevance. For oxidation axis, smoking, RFS, C-reactive protein, uric acid, hematocrit, erythrocyte sedimentation rate, albumin, white blood cell (WBC), monocyte, basophil, alpha-fetoprotein, carcinoembryonic antigen, alkaline phosphatase, aspartate aminotransferase (GOT), alanine aminotransferase (GPT), and gamma-glutamyl transferase were used. For metabolism axis, systolic and diastolic blood pressure, body mass index (BMI), waist circumference, total cholesterol, triglycerides (TG), high-density lipoprotein cholesterol (HDLC), fasting glucose were used. Age and sex were considered for both axes. We let labels of four groups as $Y\in \{\mathrm{0,1},\mathrm{2,3}\}$ and variables as $X$ that are used to make scores. Among aforementioned markers, markers that showed significant differences across different health status groups were selected using analysis of variance (ANOVA) for numerical variables and chi-squared test for categorical variables and used as predictor variables for modeling health space models. Description of the variables that are used in the model of the health spaces are described in Table 1.

**Table 1:** Detail descriptions of the predictor variables used in final health space models. KNHANES data was used to construct health spaces and Ewha-Boramae data and KARE data were used for external validation of health spaces

Data (sample size)	**Model Development**	**External Validation**	
	KNHANES (*n* = 32,140)	Ewha-Boramae (*n* = 862)	KARE (*n* = 3,199)
Age (year)	47.95 ($\pm 15.57$)	47.72 ($\pm 11.23$)	51.01 ($\pm 8.77$)
**Sex**			
Male	15,469 (48.13%)	554 (64.26%)	1,782 (55.70%)
Female	16,671 (51.87%)	308 (35.74%)	1,417 (44.29%)
**Smoking**			
Non-smokers/Past smokers	24,567 (76.44%)	690 (80.05%)	2,222 (69.46%)
Current smokers	7,573 (23.56%)	172 (19.95%)	977 (30.54%)
WBC (× 10^3^ μL)	6.19 ($\pm 1.72$)	5.87 ($\pm 1.60$)	6.63 ($\pm 1.79$)
GPT (μkat/L)	0.36 ($\pm 0.31$)	0.49 ($\pm 0.44$)	0.47 ($\pm 0.53$)
BMI (kg/m^2^)	23.68 ($\pm 3.37$)	24.13 ($\pm 3.29$)	24.54 ($\pm 3.08$)
TG (mmol/L)	1.54 ($\pm 1.30$)	1.35 ($\pm 0.78$)	1.87 ($\pm 1.18$)
HDLC (mmol/L)	1.28 ($\pm 0.31$)	1.36 ($\pm 0.33$)	1.14 ($\pm 0.25$)
Glucose (mmol/L)	5.47 ($\pm 1.29$)	5.30 ($\pm 1.03$)	4.89 ($\pm 1.26$)

Continuous variables were expressed as the mean $\pm$ standard deviation, categorical variables were expressed as frequency (percentage)

### Simulation study

A simulation study was conducted to compare the performance of three HS models. Two scenarios have been conceived in a simulation study, each of which has four sub-scenarios. We assumed there are $m$ health status groups. We considered the following parameters: total number of groups ($k$), the difference between the location parameters of the distribution of each group ($\Delta$), the common scale parameter (${\sigma }^{2}$), continuous predictor variables ($X$), discrete predictor variables (${X}^{^{\prime}}$). Continuous predictor variables $X$ and discrete predictor variables ${X}^{^{\prime}}$ can be expressed as follows:

$$X=\left({x}_{1},\cdots ,{x}_{{p}_{1}},{x}_{{p}_{1}+1},\cdots ,{x}_{{p}_{1}+{p}_{2}}\right)$$

$${X}^{^{\prime}}=\left({x}_{1}^{^{\prime}},\cdots ,{x}_{{q}_{1}}^{^{\prime}},{x}_{{q}_{1}+1}^{^{\prime}},\cdots ,{x}_{{q}_{1}+{q}_{2}}^{^{\prime}}\right)$$

The first axis of ${S}_{1}$ score is generated by ${x}_{1},\cdots ,{x}_{{p}_{1}},{x}_{1}^{^{\prime}},\cdots ,{x}_{{q}_{1}}^{^{\prime}}$ and the second axis of ${S}_{2}$ score by${x}_{{p}_{1}+1},\cdots ,{x}_{{p}_{1}+{p}_{2}},{x}_{{q}_{1}+1}^{^{\prime}},\cdots ,{x}_{{q}_{1}+{q}_{2}}^{^{\prime}}$. For the group $m\in \left\{0,\cdots ,k-1\right\} {, x}_{i}$ are randomly simulated from the normal distribution $N\left(m\Delta ,{\sigma }^{2}\right)$ and ${x}_{j}^{^{\prime}}$ are randomly simulated from the Bernoulli distribution $Bernoulli\left(\frac{m}{k+1}\right).$

For scenario 1, $\left({p}_{1},{p}_{2},{q}_{1},{q}_{2}\right)=(\mathrm{2,1},\mathrm{0,1})$; for scenario 2, $\left({p}_{1},{p}_{2},{q}_{1},{q}_{2}\right)=(\mathrm{3,2},\mathrm{1,2})$. In each sub-scenarios of scenario 1, $\Delta$ has a value of 1, 1.5, 2, and 3, and in each sub-scenarios of scenario 2, $\Delta$ has a value of 0.5, 1, 1.5, and 2. The detailed description of these scenarios is shown in Table 2.

**Table 2:** Details of simulation settings. Δ represents the difference between the location parameters of each distribution and the ${\sigma }^{2}$ represents the scale parameter of each distribution

Scenario	1				2			
Sub Scenario	1	2	3	4	1	2	3	4
$\Delta$	1	1.5	2	3	0.5	1	1.5	2
${\sigma }^{2}$	1	1	1	1	1	1	1	1
$k$	3				3			
${p}_{1}$	2				3			
${p}_{2}$	1				2			
${q}_{1}$	0				1			
${q}_{2}$	1				2			
models	Logistic regression model Proportional odds model				Logistic regression model Logistic mixed effect model Proportional odds model			

### Statistical analysis

There are several statistical models available for handling multiple categorical responses representing healthy group (coded 0), a group with one metabolic risk factor (coded 1), a group with two metabolic risk factors (coded 2), a group with metabolic syndrome or oxidative stress-related disease group (coded 3). Note that these four categories have ordered information. We first consider simple binary models focusing only on 1 and 4 categories. We considered logistic regression model and logistic mixed effect model.

Next, we consider more complex models that can handle four categories simultaneously. Candidate models included cumulative logit model [17], proportional odds model (POM) [18], and partial proportional odds model [19]. Note that cumulative logit model estimates a large number of regression coefficients, making the model overly complex. The POM assumes proportionality assumption for the cumulative logits. While this assumption is rather strong, it has the effect of simplifying the model by reducing the number of parameters. The partial POM is a model that relaxes the proportional odds assumption [19]. However, this relaxation of partial POM may often cause a discordant ordering of observed health groups and estimated health groups in HS. Thus, we do not consider the cumulative logit model and the partial proportional odds model in our analysis.

In summary, we focus on three statistical models to define the HS: logistic regression models (LRMs), Logistic mixed effects models (LMMs), and proportional odds models (POMs). From these models, we derive scores for each model and then estimate the confidence ellipses based on the F-distribution to represent the groups in the HS.

First, we considered LRM to develop HS. It is obvious that an individual with a metabolic syndrome or suffering lifestyle-related chronic diseases is in a worse health status than a healthy individual. The response variable $Y$ representing the health status of an individual is defined to be 0 for a healthy individual and 1 for an individual with a lifestyle-related chronic disease. Let $X$ represent predictor variables that are used in defining oxidation and metabolism scores such as age, sex, smoking preference, WBC, GPT, BMI, Glucose, HDLC, and TG. These predictor variables were selected by bidirectional elimination based on Akaike Information Criterion (AIC) [20]

While fitting LRM or LMM, we let health status group as $Y\in \left\{\mathrm{0,1}\right\}$ and predictor variables as $X$. The LRM is given as follows.

$$logit\left(p\right)=\alpha+X\beta,$$

where $p=P\left(Y=1\right)$ is the probability of the event $\left(Y=1\right)$. $\alpha$ is an unknown intercept parameter. $\beta$ is a vector of regression coefficients corresponding to $X$. Using the estimates of $\widehat{\alpha }$ and $\widehat{\beta }$ we let LRM score as $\widehat{\alpha }+X\widehat{\beta }$. Note that $\beta$ can be interpreted in respect to odds ratio:

The logistic mixed effect model is defined as follows

$$logit(p)=\alpha +X\beta +Z\gamma$$

where $\gamma$ represents regression coefficients corresponding to $Z$. The estimates of $\widehat{\alpha }$, $\widehat{\beta ,}$ and $\widehat{\gamma }$ can be obtained via maximum likelihood estimation [21]. We let LMM health score as $\widehat{\alpha }+X\widehat{\beta }+Z\widehat{\gamma }$. Note that $\beta$ and $\gamma$ can be interpreted in respect to the odds ratio.

In LRM and LMM, group information was not fully used, since only binary information on healthy group and unhealthy group with lifestyle-related chronic diseases were used.

To fully use other two groups’ (two groups that are in between healthy group and unhealthy group with lifestyle-related chronic diseases) information, we considered the POM which uses ordered group information from the whole group’s data. Let $Y$ represent the ordered groups. For $j=0,\cdots ,k-1,$ the cumulative probability is given by

$${\gamma }_{j}=\mathrm{Pr}\left({\varvec{Y}}\le j |\mathbf{X}\right)$$

The POM is defined in terms of ${\gamma }_{j}$ as follows,

$$logit\left(\gamma_j\right)=\alpha_j-X\beta,$$

where $X$ is a matrix of predictor variables. In terms of the POM can be repressed as follows:

$$\frac{\gamma_j}{1-\gamma_j}=exp(\alpha_j-X\beta),$$

For $k$ categories of $Y$’s, this POM estimates $(k-1$) ${\alpha }_{j}$ and only one coefficient vector $\beta$. After fitting the model, we let the score as $X\widehat{\beta }$. Note that $\beta$ can be interpreted in respect to the cumulative odds ratio.

### Health Space Index (HSI)

One of the objectives of our study is to find the most appropriate model for the HS. The traditional goodness-of-fit measures such as AIC [20] and deviance focus on the contribution of individual observations. In other words, these measures are based on deviance between each observation and its predicted values. Thus, they are not appropriate in comparing models developed for the HS, because a good model for developing HS is the one that discriminates the health status groups well.

In this regard, we developed a new measure of discrimination called Health Space Index (HSI) to find the best model among LRM, LMM, and POM. HS is developed with the scores derived from the models. For each model, there are two scores: oxidation score and metabolism score. The HS uses the oxidation score as the x-axis and the metabolism score as the y-axis. In order to calculate HSI, we first estimated the confidence ellipse for each group. The confidence ellipse is a multi-dimensional generalization of a confidence interval for one-dimension to higher dimension. In our HS we use bi-dimensional space. When the confidence ellipse is estimated, we can estimate the percentage of true classification. That is, we can estimate the proportion of the confidence ellipse of the individual’s belonging to the “true” groups.

Motivated from Jaccard index [22], a measure of similarity between data sets, we derive HSI. Note that Jaccard index is defined as

$$J\left(A,B\right)=\frac{\vert A\cap B\vert}{\vert A\cup B\vert}=\frac{\vert A\cap B\vert}{\left|A\right|+\left|B\right|-\vert A\cap B\vert},$$

where A and B are data sets.

Jaccard index has the values between 0 and 1. It has the maximum value when $A\subseteq B$ or $B\subseteq A$ and the minimum value when $A\cap B=\varnothing$. That is, Jaccard index shows how much two sets are overlapped. Therefore, Jaccard index $J\left(A,B\right)$ satisfies $0\le J\left(A,B\right)\le 1$. For a simpler comparison between different models, we propose a new measure Health Space Index (HSI). In calculating HSI, we do not compare the observed groups but rather their confidence ellipses estimated from the models.

Based on Jaccard index we propose HSI as follows. Let $({x}_{ik},{y}_{ik})$ be the ${k}^{th}$ sample of group $i$ where$i=0,\dots ,m-1 , k=1,\dots ,{n}_{i}$. Let ${f}_{i}\left(x,y\right)$ be a function of samples (${x}_{i1},{y}_{i1}),\cdots , ({x}_{i{n}_{i}},{y}_{i{n}_{i}})$ where ${f}_{i}\left(x,y\right)=0$ represents the 95% confidence ellipse constructed. Let ${a}_{i}$ be the number of samples in confidence ellipse of group$i$, defined as follows:

$$a_i=\sum\limits_{k=1}^{n_i}I(f_i\left(x_{ik},y_{ik}\right)<0)$$

In a similar way, define ${a}_{ij}$ as the number of samples of group $i$ and group $j$ in common area of confidence ellipse ${A}_{i}$ and ${A}_{j}$ as,

$${a}_{ij}=\sum\limits _{k=1}^{{n}_{i}}I\left({f}_{i}\left({x}_{ik},{y}_{ik}\right)<0\right)I({f}_{j}\left({x}_{ik},{y}_{ik}\right)<0)+\sum\limits_{l=1}^{{n}_{j}}I\left({f}_{i}\left({x}_{jl},{y}_{jl}\right)<0\right)I({f}_{j}\left({x}_{jl},{y}_{jl}\right)<0)$$

Using these ${a}_{i}$’s we define HSI as a measure of indicating how much there is an overlap between two confidence ellipse ${A}_{i}$ and ${A}_{j}$ as follows:

$$HSI\left(i,j\right)=\frac{{a}_{ij}/2}{{a}_{i}+{a}_{j}-{a}_{ij}/2}\cdot$$

A smaller value of HSI means that there is less overlap between ${A}_{i}$ and ${A}_{j}$. As most distance measures, HSI satisfies several properties.

1. $$0\le HSI\le 1$$
2. As the number of samples within the common area decreases, so does HSI.
3. HSI is a monotonically decreasing function of ${a}_{ij}$.

Furthermore, the $SMHSI=1-$ HSI satisfies semi-metric property, non-negativity, symmetry, and identity of indiscernible.

## Results

### Real data analysis

For LRMs, the predictor variables were selected by stepwise selection via AIC. Their estimates of LRMs are shown in Tables 3 and 4 for the oxidation score model and the metabolism score model, respectively. Prior to applying the LMM, age was categorized into the segment to be considered a random intercept. For the oxidation score, the categorized age variable, age_gr (age group), and sex were used as random intercepts. In defining metabolism score, sex was used as a random intercept. The coefficients of the LMM are shown in Tables 5, 6, 7, and 8. LRM included the second order interaction terms for both oxidation score and metabolism score. The coefficients of POM are shown in Tables 9 and 10 for the oxidation score model and the metabolism score model, respectively.

**Table 3:** Estimated coefficients of the oxidation score from logistic regression model

coefficients	Estimate	Std. Error	z value	Pr( >|z|)
(Intercept)	-2.69212	0.636162	-4.232	2.32E-05
age	0.063423	0.010459	6.064	1.33E-09
sex	-2.69518	0.270967	-9.947	< 2e-16
sm_presnt	-0.03549	0.153212	-0.232	0.81684
WBC	0.000454	0.072038	0.006	0.99497
GPT	-0.91637	0.689613	-1.329	0.18391
age:sex	0.029996	0.003758	7.982	1.44E-15
sex:WBC	0.158739	0.026402	6.012	1.83E-09
age:sm_presnt	-0.00561	0.002233	-2.512	0.012
WBC:GPT	0.469825	0.080383	5.845	5.07E-09
age:GPT	0.030028	0.009549	3.145	0.00166
sex:sm_presnt	0.154053	0.06537	2.357	0.01844
sm_presnt:GPT	0.226702	0.137561	1.648	0.09935
age:WBC	-0.00137	0.000936	-1.464	0.14331

**Table 4:** Estimated coefficients of the metabolism score from logistic regression model

coefficients	Estimate	Std. Error	z value	Pr( >|z|)
(Intercept)	-5.041e + 01	2.15E + 00	-23.446	< 2e-16
age	3.53E-01	1.83E-02	19.274	< 2e-16
sex	3.95E + 00	7.25E-01	5.445	5.18E-08
BMI	1.20E + 00	5.98E-02	20.047	< 2e-16
TG	5.10E + 00	7.43E-01	6.862	6.81E-12
HDLC	7.24E + 00	9.06E-01	7.987	1.38E-15
Glucose	1.92E + 00	2.58E-01	7.433	1.06E-13
age:BMI	-1.01E-02	6.97E-04	-14.544	< 2e-16
TG:HDLC	-2.118e + 00	2.03E-01	-10.417	< 2e-16
sex:HDLC	-1.403e + 00	2.09E-01	-6.714	1.90E-11
age:TG	-2.66E-02	4.77E-03	-5.573	2.50E-08
BMI:HDLC	-1.90E-01	3.51E-02	-5.403	6.56E-08
sex:Glucose	-3.43E-01	1.27E-01	-2.702	0.0069
age:sex	7.70E-03	4.26E-03	1.808	0.0705
TG:Glucose	1.90E-01	1.30E-01	1.453	0.1462

**Table 5:** The portion of the random effect of the estimated coefficients in the logistic mixed effect model of the oxidation score

**Groups**	**Name**	**Variance**	**Std.Dev**	**Corr**		
**age_gr**	(Intercept)	5.84E + 00	2.41609			
	sm_presnt	9.92E-02	0.31491	-0.95		
	WBC	1.25E-03	0.03541	-9.00E-01	0.73	
	GPT	6.77E-02	0.26016	-0.98	0.87	0.93
**sex**	(Intercept)	1.51E-01	0.38887			
	sm_presnt	9.37E-04	0.0306	-1.00E + 00		
	WBC	1.86E-03	0.04312	-1.00E + 00	1	
	GPT	8.91E-05	0.00944	1	-1	-1

**Table 6:** The portion of the fixed effect of the estimated coefficients in the logistic mixed effect model of the oxidation score

coefficients	Estimate	Std. Error	z value	Pr( >|z|)
(Intercept)	-1.64E + 00	1.12E + 00	-1.465	0.1429
sm_presnt	-2.41E-01	1.47E-01	-1.646	0.0997
WBC	3.05E-01	3.67E-02	8.313	< 2e-16
GPT	3.86E + 00	1.66E-01	23.184	< 2e-16

**Table 7:** The portion of the random effect of the estimated coefficients in the logistic mixed effect model of the metabolism score

**Groups**	**Name**	**Variance**	**Std.Dev**	**Corr**			
**sex**	(Intercept)	0	0				
	BMI	2.49E-03	0.04991	NaN			
	Glucose	3.96E-03	0.06293	NaN	-1		
	HDLC	3.69E-01	0.6072	NaN	-1	1	
	TG	5.09E-02	0.22551	NaN	1	-1	-1.00E + 0

**Table 8:** The portion of the fixed effect of the estimated coefficients in the logistic mixed effect model of the metabolism score

coefficients	Estimate	Std. Error	z value	Pr( >|z|)
(Intercept)	-17.74525	0.39072	-45.417	< 2e-16
BMI	3.27E-01	3.64E-02	8.993	< 2e-16
Glucose	2.14E + 00	7.24E-02	29.554	< 2e-16
HDLC	-2.00E + 00	4.40E-01	-4.543	5.54E-06
TG	1.94E + 00	1.69E-01	11.473	< 2e-16

**Table 9:** Estimated coefficients of the oxidation score from proportional odds model

coefficients	Estimate	Std. Error	z value	Pr( >|z|)
(Intercept):1	3.85E + 00	9.62E-02	39.992	< 2e-16
(Intercept):2	5.05E + 00	9.79E-02	51.608	< 2e-16
(Intercept):3	5.69E + 00	9.90E-02	57.458	< 2e-16
age	-6.89E-02	8.22E-04	-83.86	< 2e-16
sex	7.37E-02	3.00E-02	2.458	1.40E-02
sm_presnt	4.19E-02	1.77E-02	2.364	1.81E-02
WBC	-2.16E-01	7.21E-03	-29.994	< 2e-16
GPT	-2.42E + 00	6.08E-02	-39.767	< 2e-16

**Table 10:** Estimated coefficients of the metabolism score from proportional odds model

coefficients	Estimate	Std. Error	z value	Pr( >|z|)
(Intercept):1	1.26E + 01	1.78E-01	70.71	< 2e-16
(Intercept):2	1.43E + 01	1.83E-01	78.57	< 2e-16
(Intercept):3	1.53E + 01	1.85E-01	82.47	< 2e-16
BMI	-3.19E-01	4.72E-03	-67.59	< 2e-16
Glucose	-9.35E-01	2.20E-02	-42.4	< 2e-16
HDLC	1.91E + 00	4.65E-02	41.01	< 2e-16
TG	-7.22E-01	1.82E-02	-39.7	< 2e-16
sex	-4.20E-01	2.60E-02	-16.18	< 2e-16
age	-5.95E-02	9.03E-04	-65.95	< 2e-16

**Fig. 1:**
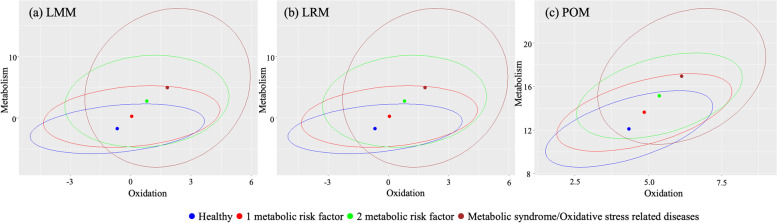
The health spaces developed from KNHANES data. **a** is health spaces made with LMM, **b** is health spaces made with LRM, and **c** is health spaces made with POM. The x-axis represents the oxidation score and y-axis represents the metabolism score. Each ellipses in different color represents the confidence region of each groups on the health space and bold dots represents the center of ellipses. Each blue, red, green, and brown color represents healthy group, 1 metabolic risk factor group, 2 metabolic risk factors group, metabolic syndrome or oxidative stress related diseases group

After making the scores using three models with the KNHANES data, we plotted the 95% confidence ellipse for each group in the two-dimensional HSs (Fig. 1-(a),(b),(c)) with the oxidation score in the x-axis and the metabolic score in the y-axis. The points in different colors mean the center of the ellipse. Blue, red, green, and brown mean healthy group (coded 0), 1-metabolic risk factor group (coded 1), 2-metabolic risk factors group (coded 2), metabolic syndrome or oxidative stress relate diseases group (coded 3), respectively. As an individual’s health condition becomes worse, the point moves to the top right of the HS.

**Fig. 2:**
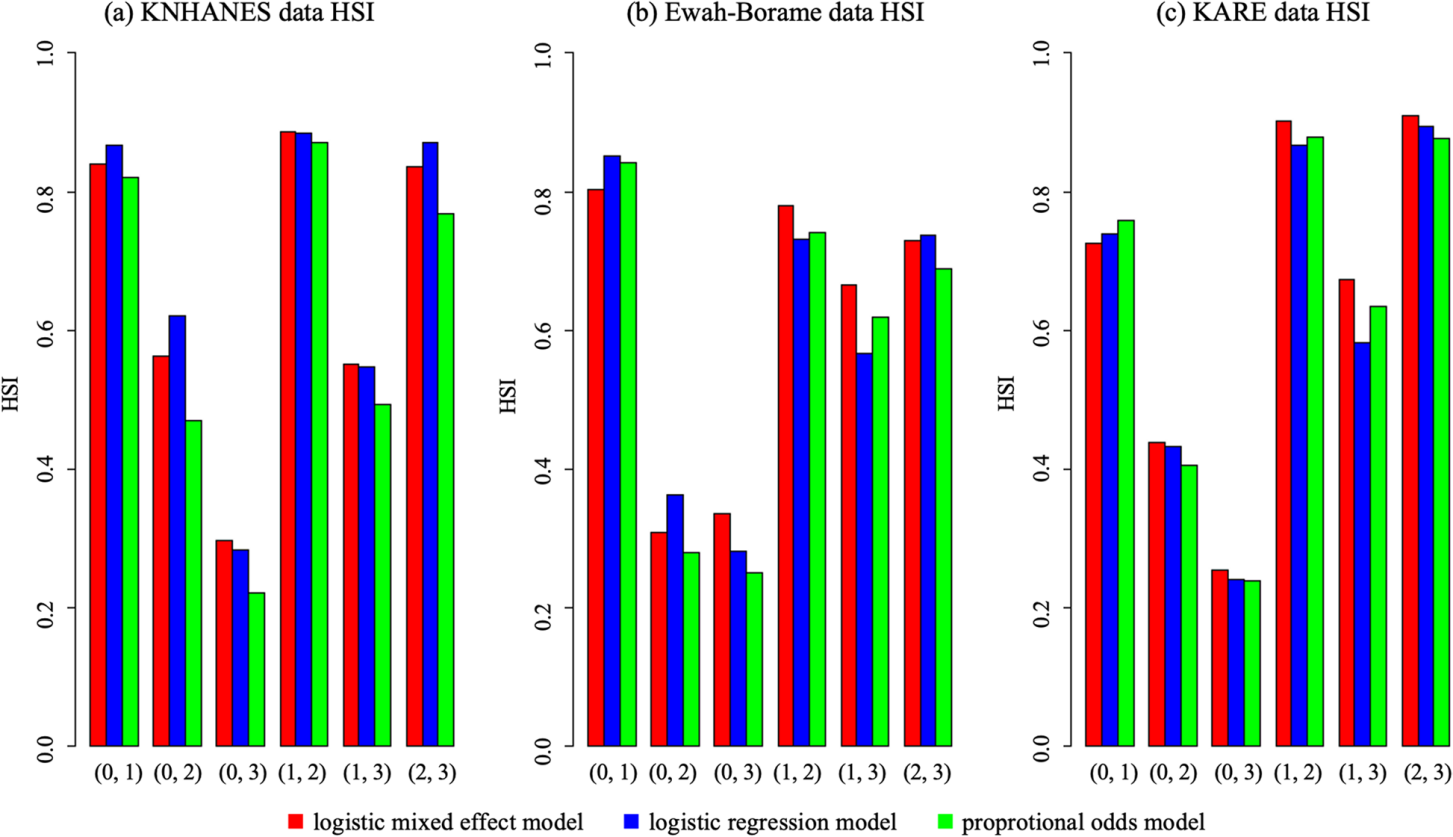
Results of validation study using KNHANES data as a training set. The x-axis represents the pair of compared groups, and the y-axis refers to the HSI. Each red, blue, and green bar represents HSI made with LMM, LRM and POM. HSI(0,3) tends to have maximum value among others and greatest with POM

To figure out how much overlaps exists between groups, we computed HSIs to compare the models. Figure 2-(a) shows all pairwise HSI between groups. For KNHANES data, HSI(0, 3) between healthy group (coded 0) and lifestyle-related chronic diseases group (coded 3) showed smaller HSIs than other pairs. Note that for HSI(0, 3) the POM had the smallest value among the three models, which holds for all other HSIs.

A validation study was conducted using two independent Ewha-Boramae cohort data and KARE data. HSs applied to Ewha-Boramae cohort data is shown in Fig. 1. (b). Like KNHANES data, HSI(0, 3) showed smaller HSIs than other pairs. Also, the POM had the smaller HSI values than other models for most pairs (Fig. 2-(b)). HSs applied to KARE data is shown in Fig. 1-(c). The same patterns were observed. That is, HSI(0, 3) showed smaller HSIs than other pairs and the POM had the smaller HSI values than other models for most pairs. (Fig. 2-(c)).

### Simulation study

**Fig. 3:**
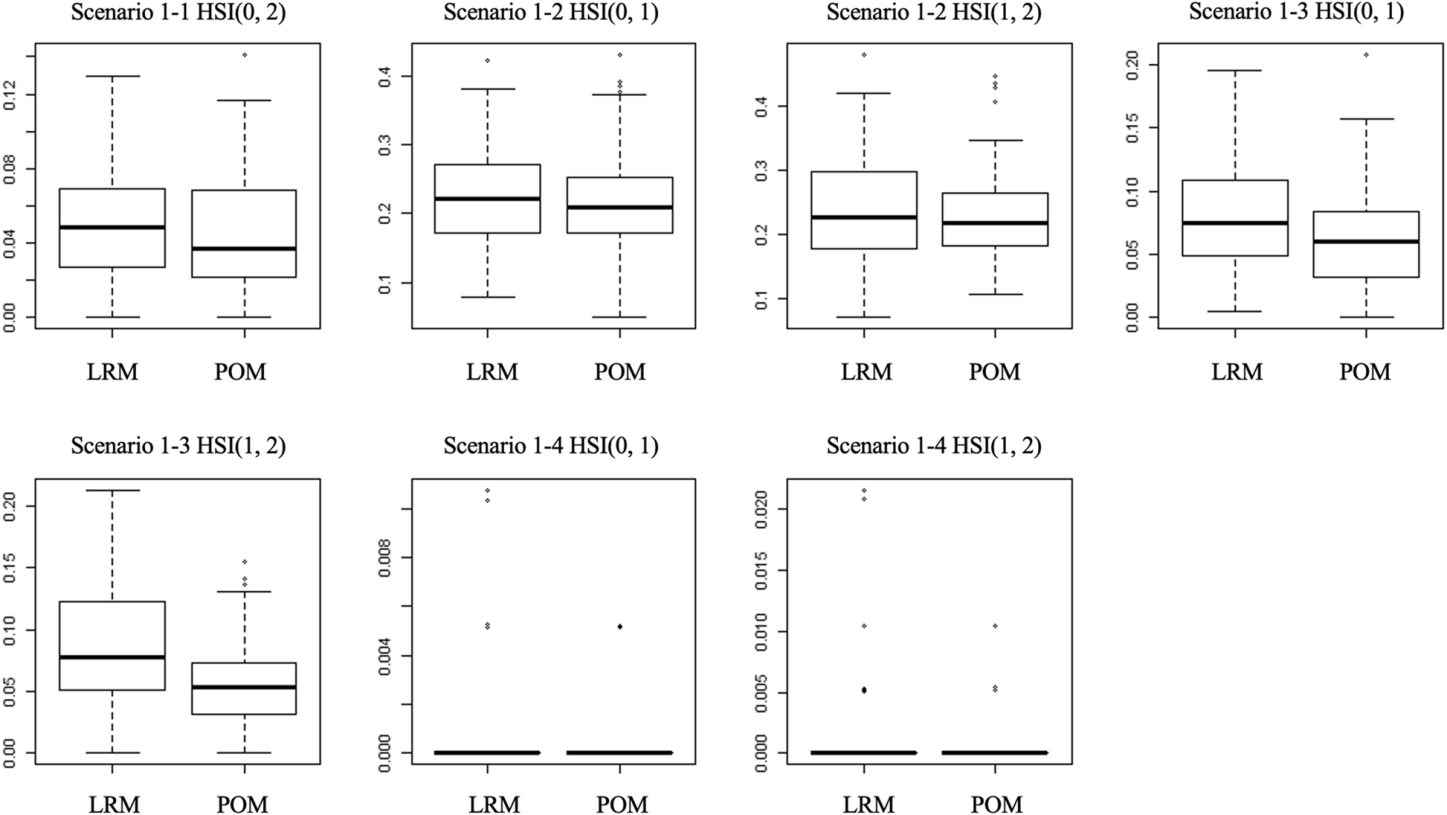
Boxplots for two models LRM and POM of scenario 1 with 50 samples. Box shows the Q1 to Q3 interquartile range and bold horizontal line show the median

**Fig. 4:**
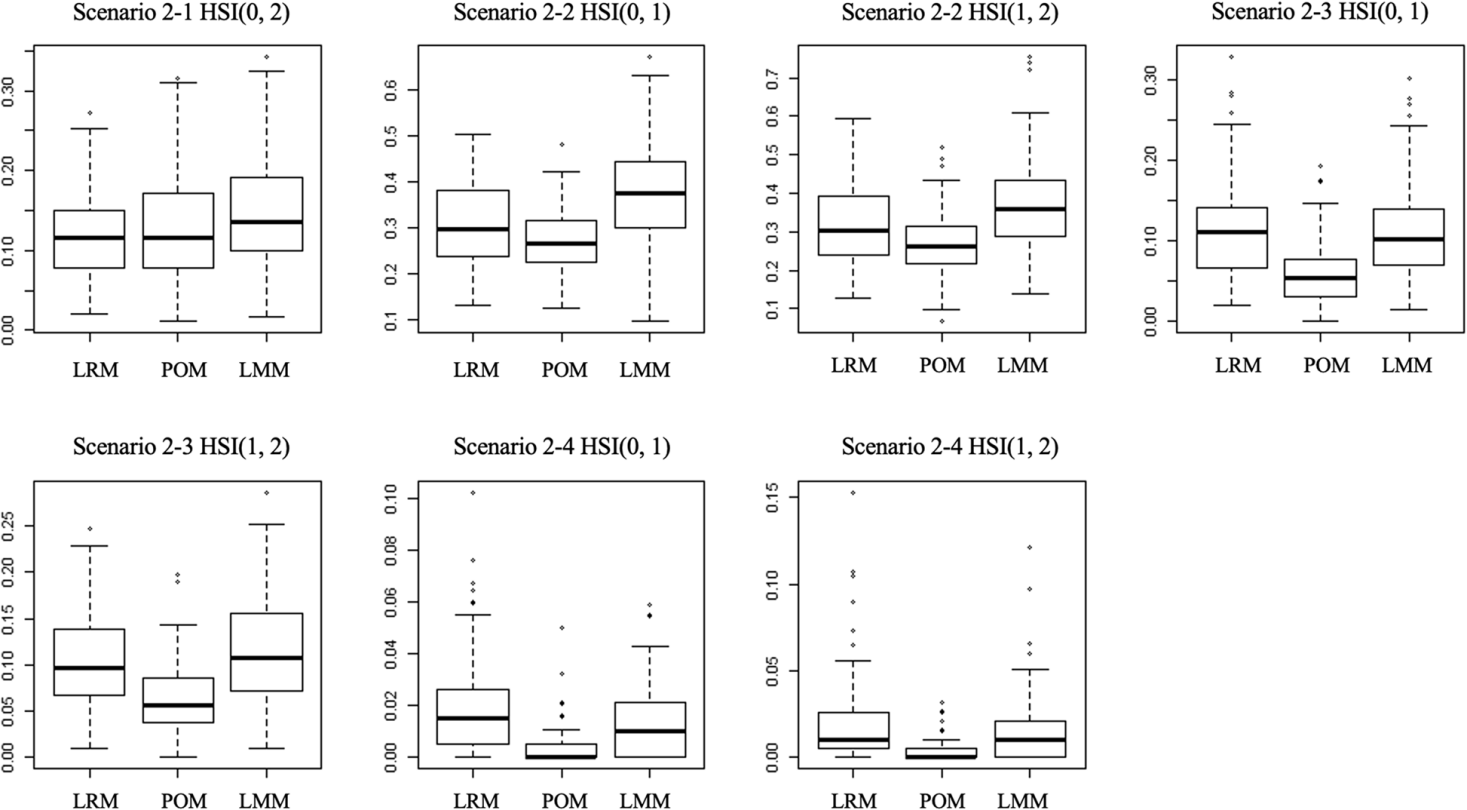
Boxplots for three models LRM, POM, and LMM of scenario 2 with 50 samples. Box shows the Q1 to Q3 interquartile range and bold horizontal line show the median

**Fig. 5:**
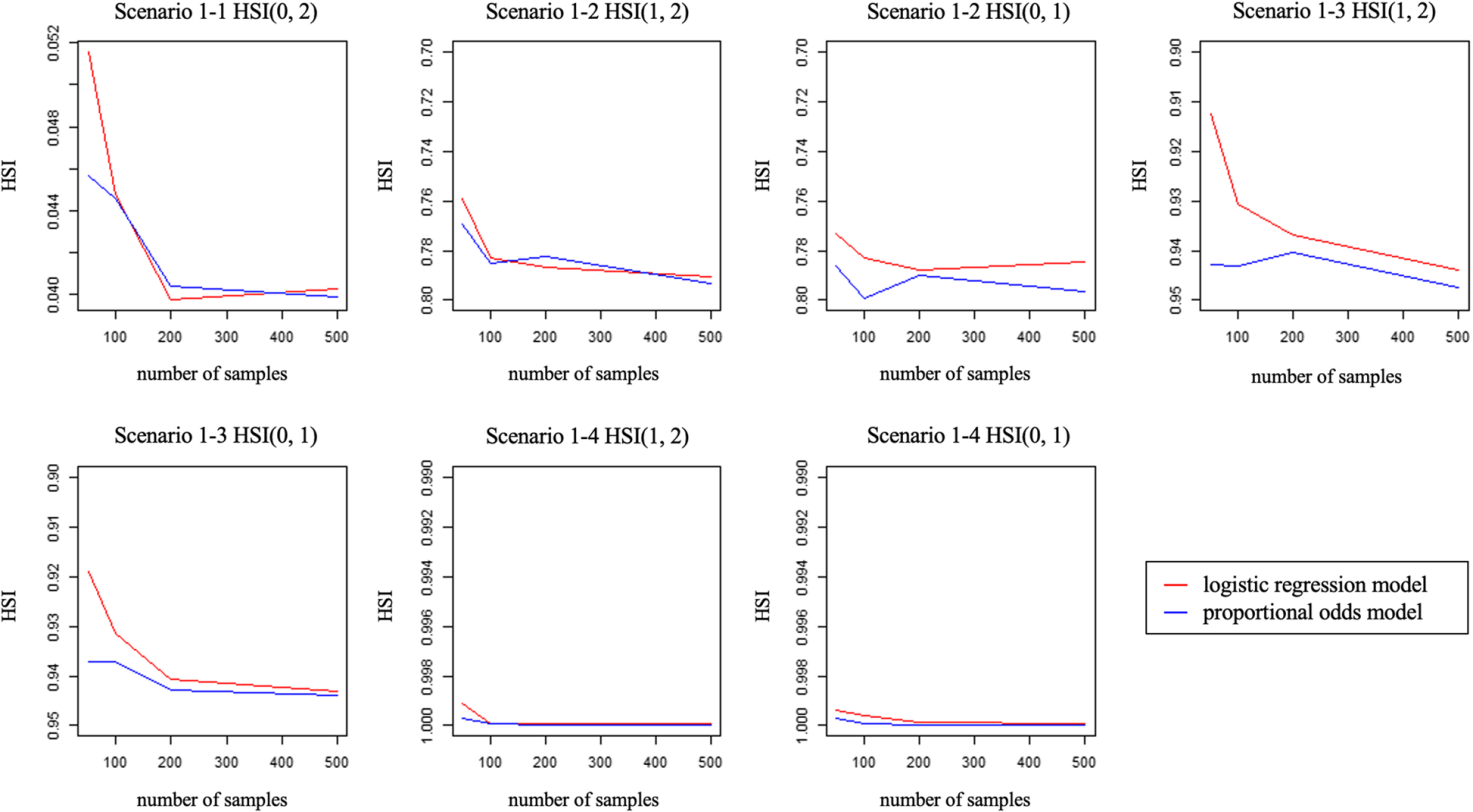
Trend graph of scenario 1. The x-axis is number of samples and y axis is corresponding HSI. Each red and blue line represents the model made by LRM and POM

**Fig. 6:**
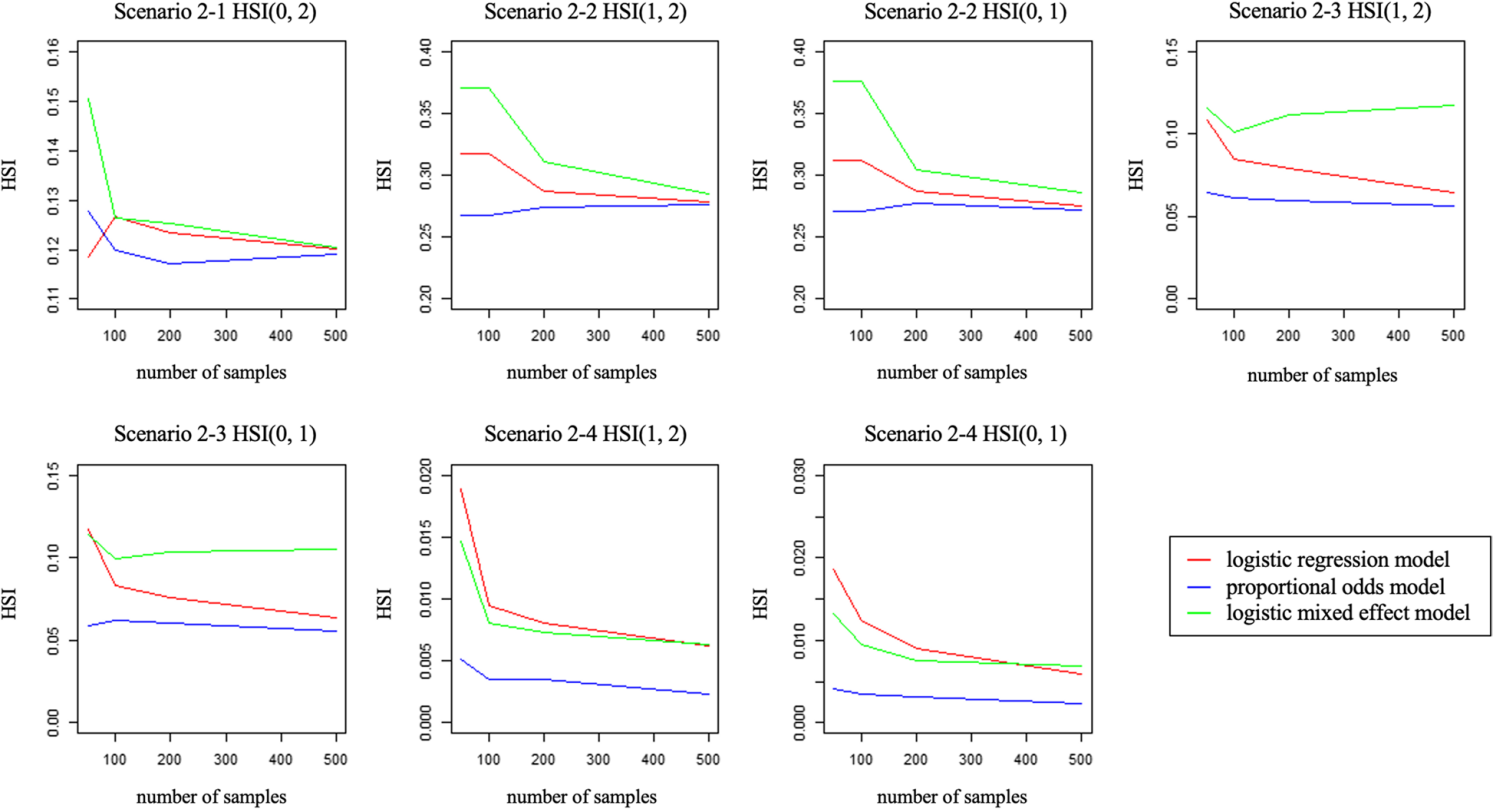
Trend graph of scenario 2. The x-axis is number of samples and y axis is corresponding HSI. Each red, blue, and green line represents the model made by LRM, POM and LMM

We compared the HSIs in the models with the boxplots (Figs. 3, 4) and trend graphs (Figs. 5, 6) of the mean of the HSI to the number of samples generated. In Scenario 1–1 and Scenario 1–2, there was no difference between the LRM and the POM, as shown in the boxplot and trend graph. In scenario 1–3, there are significant difference between LRM and POM. In Scenario 1–4, because the difference between the location parameters is too large for the scale parameters, almost all of the HSI values were zero, and there is no difference between the two models.

In Scenario 2–1 and Scenario 2–2, the HSI(0,2) in the LRM and the POM was similar, but in the LMM it had a value larger than the previous two models. In Scenario 2–3 and Scenario 2–4, the HSI(0,1) and HSI(1,2) in the POM were smaller than those of LRM and LMM.

## Discussion

We presented that POM outperformed LRM and LMM in discriminating different health groups in terms of oxidative and metabolic stresses not only in the simulation, but also in the Korean general adult population. The previous HSs [7] were based on the small sample sizes simply including axes and points and were only referring to approximate differences between groups. On the other hand, our HS is based on large sample size and uses the more systematically derived statistical models. Furthermore, we validated our result using the data from two different independent population studies: the Ewha-Boramae cohort [13] and the KARE data [14]. This indicates that individual’s health condition positioned on the HS can be distinctive from the others in terms of oxidative and metabolic stresses. Our finding also suggests that the two-dimensional HS might enable to distinguish different health status of target individuals from healthy individuals: i.e., subjects at risk having metabolic risk or lifestyle-related chronic diseases.

We estimated the confidence ellipses of each group and visualized them in HS. By quantifying how much they are overlapped on basis of the HSI, we compared the performance of HS created using different statistical models. The simulation study indicated that the POM model tended to have the smallest index among three models and outperformed on differentiating the target risk groups from the healthy group. Furthermore, in each data, except in LRM for Ewha-Boramae cohort data, HSI (0,3) in the HS from POM takes the smallest values among all the other HSIs’, indicating that the HS of POM performed best.

Our findings are consistent with the literature regarding the significance of components in the both axes for predicting lifestyle-related chronic diseases and their outcomes. It was reported that the significant predictor variables for mortality in older adults with diabetes included age, gender, smoking status, BMI, fasting glucose, WBC, and GPT [23]. A role of smoking status was also shown in predicting mortality outcomes, in particular for cardiovascular mortality [24]. In addition, GPT, WBC, HDL, TG, and fasting glucose were presented as significant components for cardiovascular outcomes including stroke prediction [25, 26]. WBC might serve as a potential predictor for type 2 diabetes, hypertension [27], and atherosclerosis in the patients with metabolic disorders [28]. The Asian diabetic risk score was developed including age, gender, smoking status, BMI, fasting plasma glucose, HDL-cholesterol and TG [29]. Another risk-prediction model for new-onset hypertension included age, sex, BMI, and smoking status [30]. These models were suggested to form the foundation of personalized healthcare system [25]. Likewise, our HS model may also be implemented for decision making in personalized healthcare.

The strengths of the present study include the utilization of comprehensive clinical data from the general population. However, there are several limitations that warrant discussion. We examined cross-sectional data, which limits the ability to infer causal relationship between the predictor variables and lifestyle-related chronic diseases. Study population is representative of the age spectrum of the entire adult population in South Korea, but which may limit the generalizability to other populations. The presented HS was built through classical logistic regression models. Further consideration of data mining algorithms is also needed such as support vector machines, k-nearest neighbors algorithm, and deep learning to improve the classification accuracy. Our finding also warrants further prospective evaluation to determine whether the suggested HS model can be utilized as a prognostic model for predicting the onset of lifestyle-related chronic diseases.

The result is in line with the idea that a composite biomarker may enable better monitoring of disease progression as compared to single measures [31]. Since our model considered the interrelationships of multiple markers, it may help to improve the prediction of disease progression, which is complex multidimensional biological systems. It may also help avoid erroneous conclusions and provide effective summative evaluation of individual’s health outcome [31]. More importantly, a prediction model needs to provide accurate and validated estimates of probabilities of specific health conditions or outcomes in the targeted individuals [32]. Building a model based on affordable and easily obtainable clinical data could improve a major public health problem using a quick, simple, and inexpensive approach that is both safe and acceptable to the target population.

## Conclusions

HS model is an effective way to visualize individual’s health status in an objective way. Through empirical studies, we successfully validated the usefulness of our proposed HS model using two independent datasets. Our HS model might show a great promise in encouraging behavioral change and improving healthy lifestyles or reducing risk factors. This suggests that the presented HS model may not only potentially be used to stratify individuals at risk having metabolic risk or lifestyle-related chronic diseases, but also help the individuals to perceive their health status and to engage in empowered way.

## Supplementary Information

**Additional file 1: **Proof of properties of HSI.

## Data Availability

The KNHANES and KARE datasets can be provided after review and evaluation of research plan by the Korea Centers for Disease Control and Prevention (http://www.cdc.go.kr/CDC/eng/main.jsp). The Ewha-Boramae dataset is available from the corresponding author on reasonable request.

## Abbreviations

HS: Health Space
HSI: Health Space Index
KNHANES: Korea National Health And Nutrition Examination Survey
LRM: Logistic regression model
LMM: Logistic mixed effects model
POM: Proportional odds model
