# Supplementary material for: Statistical modeling of health space based on metabolic stress and oxidative stress scores
Source: BMC Public Health. 2022 Sep 8;22:1701. doi: 10.1186/s12889-022-14081-0 (PMC9454208; doi:10.1186/s12889-022-14081-0)
Supplement: Supplementary file 1 — Additional file 1: Proof of properties of HSI. [file 12889_2022_14081_MOESM1_ESM.docx]

**Appendix 1**

**Proof of properties of HSI**

(1)

$HSI(i,j)=\frac{\frac{a_{ij}}{2}}{a_{i}+a_{j}-\frac{a_{ij}}{2}}\leq1\Leftrightarrow\frac{a_{ij}}{2}\leq a_{i}+a_{j}-\frac{a_{ij}}{2} \Leftrightarrow a_{ij}\leq a_{i}+a_{j}$ , HSI$\leq1$ is trivial

(2)

Let $f\left( a_{ij} \right)=\frac{a_{ij}/2}{a_{i}+a_{j}-a_{ij}/2}$ for fixed $a_{i}$ and $a_{j}$ then, $f^{'}\left( a_{ij} \right)=\frac{2\left( a_{i}+a_{j} \right)}{\left\{ 2\left( a_{i}+a_{j} \right)-a_{ij} \right\}^{2}}0$ so for fixed $a_{i}$ and $a_{j}$ , when number of points in common area $a_{ij}$ increase, HSI increase.

(3)

For $p,q,r,s\in\{1,2,\cdots,n\},$ suppose that $a_{p}+a_{q}=a_{r}+a_{s}$. Then when if there are more points in common area of $A_{p}$ and $A_{q}$ than $A_{r}$ and $A_{s}$ , it is obvious that $\frac{a_{rs}}{a_{r}+a_{s}}<\frac{a_{pq}}{a_{p}+a_{q}}$ holds.

Now, when $a_{p},a_{q},a_{r} and a_{s}$ are all different, we want to show if there is more points in common area, HSI also get smaller.

Let $\alpha_{ij}=\frac{a_{ij}}{a_{i}+a_{j}}$ then $HSI(i,j)$=$g\left( \alpha_{ij} \right)=\frac{\frac{a_{ij}}{2}}{a_{i}+a_{j}-\frac{a_{ij}}{2}}=\frac{\frac{\alpha_{ij}\left( a_{i}+a_{j} \right)}{2}}{a_{i}+a_{j}-\frac{\alpha_{ij}\left( a_{i}+a_{j} \right)}{2}}=\frac{\alpha_{ij}}{2-\alpha\_ij}$ and since $g^{'}\left( \alpha_{ij} \right)=\frac{2}{\left( 2-\alpha_{ij} \right)^{2}}0$, we show that HSI is monotonically increasing function of $\alpha_{ij}$.

**Appendix 2**

**Proof of properties of semi-metric space of SMHSI**

I). Non-negativity

We show that $0\leq HSI\leq1$ so its trivial that $0\leq1-HSI=SMHSI$

II). Symmetry

$$HSI\left( i,j \right)=1-\frac{\frac{a_{ij}}{2}}{a_{i}+a_{j}-\frac{a_{ij}}{2}}=1-\frac{\frac{a_{ji}}{2}}{a_{j}+a_{i}-\frac{a_{ji}}{2}}=HSI(j,i)$$

So symmetry also holds.

III). Identity of indiscernibles

Identity of indiscernibles is that HSI$\left( i,j \right)=0$ holds if and only if $i=j$.

$$HSI\left( i,j \right)=0 \Leftrightarrow1-\frac{\frac{a_{ij}}{2}}{a_{i}+a_{j}-\frac{a_{ij}}{2}}=0 \Leftrightarrow a_{ij}=a_{i}+a_{j}$$

$$\Leftrightarrow\sum_{k=1}^{n_{i}} I\left( f_{i}\left( x_{ik},y_{ik} \right)<0 \right)I\left( f_{j}\left( x_{ik},y_{ik} \right)<0 \right)+\sum_{l=1}^{n_{j}} I\left( f_{i}\left( x_{jl},y_{jl} \right)<0 \right)I\left( f_{j}\left( x_{jl},y_{jl} \right)<0 \right)=\sum_{k=1}^{n_{i}} I\left( f_{i}\left( x_{ik},y_{ik} \right)<0 \right)+\sum_{l=1}^{n_{j}} I\left( f_{j}\left( x_{jl},y_{jl} \right)<0 \right)$$

$$\Leftrightarrow\sum_{k=1}^{n_{i}} I\left( f_{i}\left( x_{ik},y_{ik} \right)<0 \right)\left( 1-I\left( f_{j}\left( x_{ik},y_{ik} \right)<0 \right) \right)+\sum_{l=1}^{n_{j}} I\left( f_{j}\left( x_{jl},y_{jl} \right)<0 \right)\left( 1-I\left( f_{i}\left( x_{jl},y_{jl} \right)<0 \right) \right)=0$$

$$\Leftrightarrow\sum_{k=1}^{n_{i}} I(x_{ik}\in A_{i})(1-I\left( x_{ik}\in A_{j} \right))=0 , \sum_{l=1}^{n_{j}} I(x_{jl}\in A_{j})(1-I(x_{jl}\in A_{i}))=0 (*)$$

$since all terms, I\left( f_{i}\left( x_{ik},y_{ik} \right)<0 \right)\in\left\{ 0,1 \right\} so all I\left( f_{i}\left( x_{ik},y_{ik} \right)<0 \right)\left( 1-I\left( f_{j}\left( x_{ik},y_{ik} \right)<0 \right) \right)\in\left\{ 0,1 \right\}$ means that $\sum_{k=1}^{n_{i}} I(f_{i}\left( x_{ik},y_{ik} \right)<0)(1-I\left( f_{j}\left( x_{ik},y_{ik} \right)<0 \right))\geq0$. Also, because that all $I\left( f_{i}\left( x_{ik},y_{ik} \right)<0 \right)\left( 1-I\left( f_{j}\left( x_{ik},y_{ik} \right)<0 \right) \right)$ takes values from $\{0,1\}$, all the terms must be 0 to hold $(*)$. It means that when $f_{i}\left( x_{ik},y_{ik} \right)<0$ , it must be $f_{j}\left( x_{ik},y_{ik} \right)<0$ and when $f_{j}\left( x_{jl},y_{jl} \right)<0$ then $f_{i}\left( x_{jl},y_{jl} \right)<0$ also holds.

So, HSI$\left( a_{i},a_{j} \right)=0 if and olny if i=j$ has been proved.

IV). There is counter example of triangular inequality. Think about three groups and its confidence ellipse for $i,j,k\in\{1,2,,,n\}$. Suppose following conditions holds.

$$1. \forall u\in\left\{ 1,\ldots,n_{i} \right\}, f_{i}\left( x_{iu},y_{iu} \right)<0 then f_{k}\left( x_{iu},y_{iu} \right)<0$$

$$2. \forall v\in\left\{ 1,\ldots,n_{j} \right\}, f_{j}\left( x_{jv},y_{jv} \right)<0 then f_{k}\left( x_{jv},y_{jv} \right)<0$$

$$3. \forall u\in\left\{ 1,\ldots,n_{i} \right\}, f_{i}\left( x_{iu},y_{iu} \right)<0 then f_{j}\left( x_{iu},y_{iu} \right)>0$$

$$4. \forall v\in\left\{ 1,\ldots,n_{j} \right\},f_{j}\left( x_{jv},y_{jv} \right)<0 then f_{i}\left( x_{jv},y_{jv} \right)>0$$

Let $a_{i}=a_{j}=20,a_{k}=10$, $\sum_{t=1}^{10} I\left( f_{i}\left( x_{kt},y_{kt} \right)<0 \right)I(f_{k}\left( x_{kt},y_{kt} \right)<0)=5$,

$\sum_{t=1}^{10} I\left( f_{j}\left( x_{kt},y_{kt} \right)<0 \right)I\left( f_{k}\left( x_{kt},y_{kt} \right)<0 \right)=5$. Then $a_{ik}=25, a_{jk}=25, and a_{ij}=0$.

Now, $HSI\left( i,j \right)=1-\frac{0}{40}=1$, $HSI\left( i,k \right)=1-\frac{12.5}{30-12.5}=\frac{5}{17.5}=HSI\left( j,k \right)$ and

$HSI\left( i,j \right)>HSI\left( i,k \right)+HSI(k,j)$ so, triangular inequality doesn’t holds.
